# Surgery

**Published:** 1896-06

**Authors:** 


### SURGERY.

EDITED BY FLOYD W. MCRAE. M.D.

### THE ACTION OF ETHER AND CHLOROFORM ON THE KIDNEY.

From a critical analytical study of 130 cases of narcosis in which the urine was examined prior to the operation and from three to six days after operation, both by chemical analysis and microscopical examination, Eisendrath draws the following conclusions:

1. Albuminuria, which is already present, is increased more frequently by ether than by chloroform.

2. Albuminuria makes its appearance, however, more frequently after chloroform-narcosis than after ether-narcosis—that is, in the proportion of 32 to 25.

3. Their action is equal upon amyloid disease of the kidney.

4. Tube-casts, with and without albumin, are found with equal frequency after chloroform- and ether-narcosis, but disappear more rapidly in the case of ether. This harmful late action of chloroform upon the kidney has been proven to be greater than that of ether by E. Fraenkel.

The author reports in detail an interesting case of death following chloroform narcosis, in which the pathological lesion was a necrosis of the parenchymatous cells of the kidney.—*American Journal Medical Science.*

---

### CIRCUMSTANCES UNDER WHICH CHLOROFORM IS PREFERABLE TO ETHER AS AN ANESTHETIC.

Gay, in commenting on anesthetics (*Boston Med. and Surg. Journal,* 1895, No. 18), says: "Sulphuric ether, under ordinary conditions, is as safe as can reasonably be expected of any agent which produces such beneficent results; that chloroform is safe in a vast majority of instances, but that fatal accidents do occasionally occur under circumstances under which they were not anticipated, and that these accidents can neither be foreseen, prevented, nor remedied; that under rare conditions no known anesthetic is entirely free from danger; that the greater comparative safety of sulphuric ether is slowly being recognized, and hence that its use is gradually displacing, to a certain extent, its more potent and more dangerous rival." The relative safety of these two agents, as just expressed, the writer thinks is decided as surely and as firmly by an intelligent and searching experience of half a century as it is possible to decide any scientific point.

As is well known, however, chloroform is to be preferred for certain cases. These the author indicates in a general way as follows: "All operations liable to be attended with spasms of the glottis, edema of the larynx or lungs, profuse secretion of fluids in the air-passages, and chronic contraction of the muscles of respiration; all cases requiring tracheotomy and esophagotomy, as membranous croup; laryngitis, acute, chronic, traumatic, specific, or tubercular; edema of the larynx or glottis; malignant disease of the throat and neck; deep cervical cellulitis; deep tumors of the neck, as bronchocele; foreign bodies in the air passages; foreign bodies in the esophagus; bronchitis in the aged; asthma; and, finally, in cases liable to be complicated by difficult or suspended respiration. —*American Journal Medical Sciences.*

---

### CANCER OF THE BREAST.

Dr. W. L. Rodman states that the results of Keen, Bull, Dennis, Weir, Halstead, and Powers, six Americans, who have within the year published their statistics in operation for cancer of the breast, show a mortality of less than one per cent. (six hundred and fifty-six operations and six deaths). He concludes his paper with the following propositions:

First. All mammary growths should be removed at once, for innocent tumors carried for a long time become a menace.

Second. The complete operation should always be done in cases of malignant disease.

Third. In nearly every case it is simply impossible to detect enlarged glands until the axilla is opened. Keen says that he cannot do so once in ten times.

Fourth. The mortality should be, with average operators, about three per cent.

Fifth. A radical operation should promise from twenty-five to fifty per cent. of permanent cures, according to the time when patients apply.

Sixth: When in doubt, operate; never wait for symptoms.—*Medical Record.*

---

### APPENDICITIS.

Dr. W. Meyer (*Med. Rec.*, Feb. 29,) sums up the indications for operative intervention as follows:

1. In cases of diffuse perforative appendicitis the operation must always be done at once. Patients have the best chance to recover if operated upon within the first twelve hours. Exceptionally patients get well without an operation.

2. In cases of acute appendicitis the patients always need careful observation. If the pulse goes up above 116 to 120 and has the tendency to stay there, the indication for an operation is given.

In case of doubt the operation is better than waiting.

3. In cases of sub-acute (mild) attack of appendicitis, also after the first severe attack from which the patient recovers without immediate operation, the appendix should be removed. The appendix, once inflamed, has to be looked upon as a diseased organ which is very apt to give repeated and more serious, even fatal, trouble in the future.—*International Journal of Surgery.*

---

### ANESTHESIA STATISTICS.

The following statistics are obtained from a collective investigation undertaken by the German Surgical Society (Gurlt), covering the five years, 1890–95. In this time chloroform was administered 201,224 times, with 88 deaths, or one in 2,286; ether 42,141 times, with 7 deaths, or one in 6,020; mixture of alcohol, chloroform, and ether 5,744 times, with one death; mixture of chloroform and ether 10,162 times, with one death. This report is surprisingly favorable to "mixed anesthesia."

